# IFITM proteins are incorporated onto HIV-1 virion particles and negatively imprint their infectivity

**DOI:** 10.1186/s12977-014-0103-y

**Published:** 2014-11-25

**Authors:** Kevin Tartour, Romain Appourchaux, Julien Gaillard, Xuan-Nhi Nguyen, Stéphanie Durand, Jocelyn Turpin, Elodie Beaumont, Emmanuelle Roch, Gregory Berger, Renaud Mahieux, Denys Brand, Philippe Roingeard, Andrea Cimarelli

**Affiliations:** CIRI, Centre International de Recherche en Infectiologie, Lyon, F69364 France; INSERM, U1111, 46 Allée d’Italie, Lyon, F69364 France; Ecole Normale Supérieure de Lyon, 46 Allée d’Italie, Lyon, F69364 France; CNRS, UMR5308, 46 Allée d’Italie, Lyon, F69364 France; University of Lyon, Lyon I, UMS3444/US8 BioSciences Gerland, Lyon, F69364 France; Plateforme des Microscopies, PPF ASB, Université F. Rabelais et CHRU de Tours, Tours, France; INSERM U966, Université F. Rabelais et CHRU de Tours, Tours, France; Present address: Department of Infectious Diseases, King’s College London School of Medicine, London, SE1 9RT UK

**Keywords:** HIV, IFITMs, Restriction factors, Interferon

## Abstract

**Background:**

Interferon induced transmembrane proteins 1, 2 and 3 (IFITMs) belong to a family of highly related antiviral factors that have been shown to interfere with a large spectrum of viruses including Filoviruses, Coronaviruses, Influenza virus, Dengue virus and HIV-1. In all these cases, the reported mechanism of antiviral inhibition indicates that the pool of IFITM proteins present in target cells blocks incoming viral particles in endosomal vesicles where they are subsequently degraded.

**Results:**

In this study, we describe an additional mechanism through which IFITMs block HIV-1. In virus-producing cells, IFITMs coalesce with forming virions and are incorporated into viral particles. Expression of IFITMs during virion assembly leads to the production of virion particles of decreased infectivity that are mostly affected during entry in target cells. This mechanism of inhibition is exerted against different retroviruses and does not seem to be dependent on the type of Envelope present on retroviral particles.

**Conclusions:**

The results described here identify a novel mechanism through which IFITMs affect HIV-1 infectivity during the late phases of the viral life cycle. Put in the context of data obtained by other laboratories, these results indicate that IFITMs can target HIV at two distinct moments of its life cycle, in target cells as well as in virus-producing cells. These results raise the possibility that IFITMs could similarly affect distinct steps of the life cycle of a number of other viruses.

**Electronic supplementary material:**

The online version of this article (doi:10.1186/s12977-014-0103-y) contains supplementary material, which is available to authorized users.

## Background

The interferon-induced transmembrane proteins (IFITMs) are a family of highly related proteins composed of 5 members in humans (IFITM1, −2, −3, −5 and −10) [1,2]. Of these, IFITM1, −2 and −3 have emerged as broad-acting restriction factors capable of interfering with the replication of a number of viruses including Filoviruses, Coronaviruses, Influenza virus, Dengue virus and the type 1 human immunodeficiency virus (HIV-1) [3-12]. To stress the importance that IFITMs play in the control of viral infection, IFITM3 knockout mice display increased mortality and viral burden following influenza A virus challenge [8,13] and a specific polymorphism in the IFITM3 allele has been associated to increased susceptibility to influenza virus infection in humans [13,14].

At present, IFITMs have been described to welcome incoming viral particles and retain them into endosomal vesicles, where they are subsequently degraded [7,9-11,15-21]. Interestingly, this mechanism of inhibition seems to be active against both pH-dependent and -independent viruses that require or not the low pH of endosomes to trigger viral-to-cell membrane fusion [22,23]. The finding that pH-independent viruses can also functionally access the cytosol from endosomal vesicles likely explains the broad antiviral effects of IFITMs against these diverse classes of virus [24].

IFITMs have been proposed to block hemifusion, the process whereby the outer, but not the inner, leaflet of the viral and cellular membranes merge [17], possibly through the modulation of the intracellular levels of cholesterol, a particular intriguing hypothesis given that IFITM3 interacts with the vesicle-membrane-protein-associated protein 1 (VAPA), a key component in cholesterol homeostasis [25]. The exact mechanism of antiviral inhibition by IFITMs remains however unclear, given that a recent study indicated that IFITM3 inhibits the transition from hemifusion to pore formation rather than hemifusion itself, in a cholesterol-independent manner [26].

Given that IFITMs are typical interferon-stimulated genes (ISGs) and that the signature of a broad antiviral type I interferon response accompanies HIV-1 replication both *in vivo* and *ex vivo* [27,28], we reasoned that IFITMs may be present not only in target cells, but also in virus-producing cells during the *de novo* assembly of virion particles. Therefore, we explored the role that IFITMs may play in HIV-1 producing cells. The results we have obtained indicate that IFITMs coalesce with the structural protein Gag and are incorporated into HIV-1 viral particles both in established cell lines, as well as in primary human monocyte-derived macrophages (MDM). Virions incorporating IFITMs display decreased infectivity when compared to HIV-1 *wild type* particles in single round infection assays and a similar inhibition is observed for different retroviruses and Envelope (Env) pseudotypes. Virions incorporating IFITMs display a defect at the step of viral entry into target cells that well correlates with the infectivity defect measured here.

In conclusion, our results together with existing data in the literature indicate that IFITM proteins interfere with HIV-1 replication at two steps of the viral life cycle; in target cells by retaining incoming particles into endosomes and in virus-producing cells, by leading to the production of virions of decreased infectivity. This dual mechanism of inhibition may be similarly exerted against other viruses.

## Results

### The ectopic expression of IFITMs in HIV-1-producing cells diminishes the infectivity of viral particles

**Figure 1:**
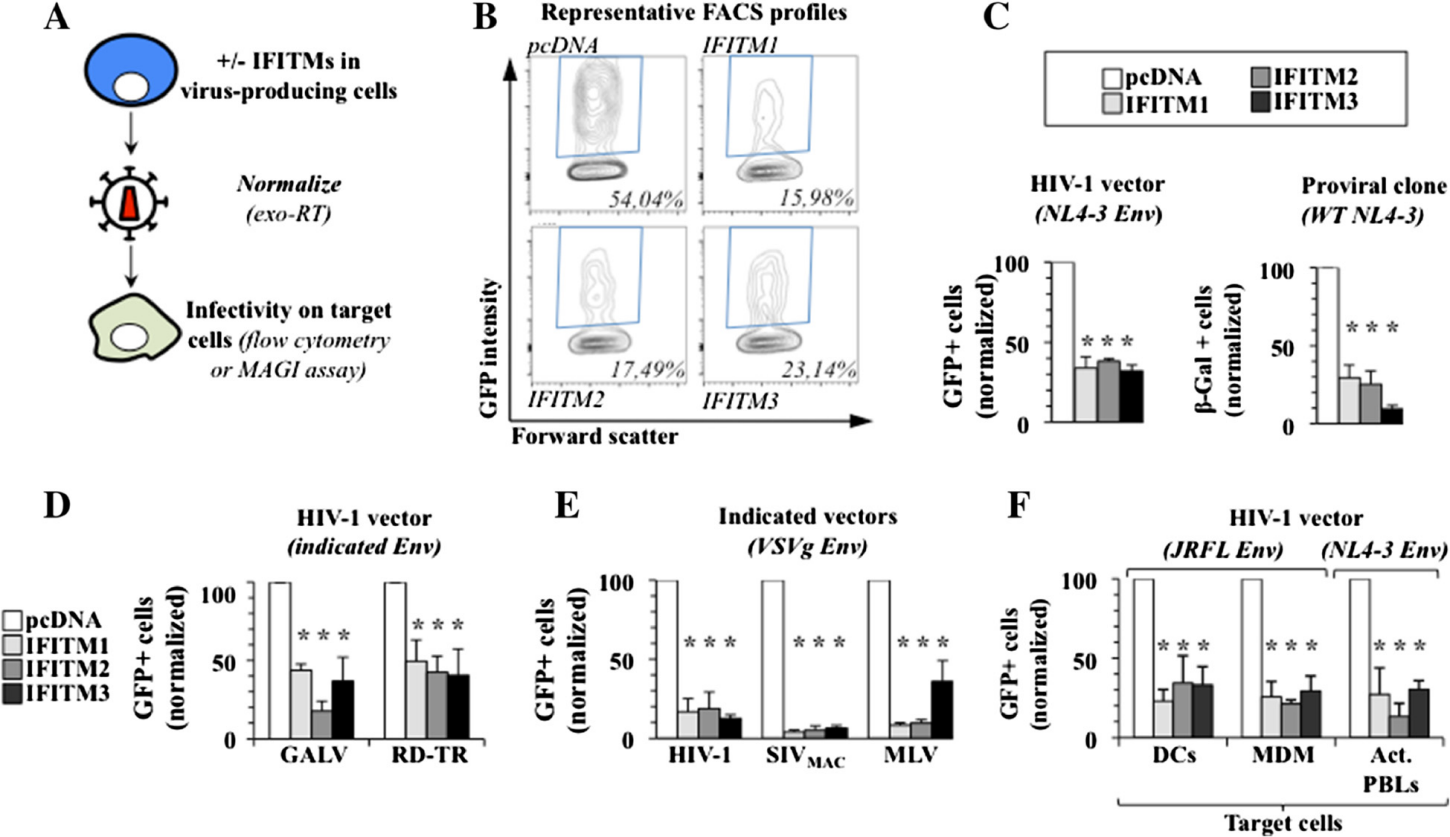
**Expression of IFITMs in virus producing cells affects the production of infectious HIV-1 viral particles. A)** Representation of the experimental scheme used here. HEK293T cells were transiently transfected with DNAs coding for single round infection-competent HIV-1 expressing GFP along with DNAs coding Flag-IFITMs. Virions were purified by ultracentrifugation through a 25% sucrose cushion, normalized by exogenous-RT (exo-RT) and used to challenge HeLaP4 or HEK293T cells bearing the appropriate HIV-1 receptors. Viral infectivity was measured 3 days later by flow cytometry in the case of GFP-coding viruses or 24 hours later by β-gal assay in the case of a complete NL4-3 proviral DNA (thanks to the HIV-1 LTR-β-gal reporter integrated in HeLaP4 cells). **B)** Typical FACS profiles obtained after this procedure. **C)** Normalized infectivity of viral particles obtained after flow cytometry analysis (left graph) or MAGI assay (right graph). **D)** As above, except that HIV-1 viruses coding GFP were pseudotyped with the indicated envelope proteins. **E)** As above, except that distinct retroviral vectors pseudotyped with VSVg and coding GFP were analyzed. **F)** As above, except that HIV-1 vectors bearing the indicated envelope were used to challenge the indicated target cells prior to flow cytometry analysis 3 days after infection. DCs and MDM were obtained after differentiation of primary human blood monocytes in GM-CSF/IL4 or M-CSF for 4 to 6 days. PBLs were activated with PHA/IL2 for 24 hours prior to viral challenge. All graphs present averages and SEM obtained from 4 to 6 independent experiments. *indicates statistically significant differences between WT and IFITMs conditions after a Student *t* test: p ≤ 0,05.

To determine whether they could affect the production of infectious HIV-1 viral particles, N-terminal Flag-tagged IFITMs were ectopically expressed along with DNAs coding for single round infection-competent HIV-1 viruses in HEK293T cells, according to the scheme depicted in Figure 1A. The transfected DNAs coded the HIV-1 Gag-Pol plus non-structural viral proteins, the indicated envelope, as well as a miniviral genome bearing a GFP expression cassette, except where a complete provirus was used, as indicated. Two days after transfection, supernatants were pre-cleared by centrifugation at low speed, then by filtration through a 0,45 μm syringe filter and were lastly purified by ultracentrifugation through a 25% sucrose cushion. Under these conditions, expression of IFITMs induced only a minor defect of virus production, as quantified by exogenous-RT activity (exo-RT, Additional file 1: Figure S1A). To focus solely on the infectivity defect of retrieved viral particles, virions were normalized by exo-RT and then used to challenge either HeLaP4 cells stably expressing the HIV receptor/co-receptor CD4/CXCR4, prior to flow cytometry 3 days afterwards (a typical FACS profile is presented in Figure 1B). Virions produced in the presence of the different IFITMs displayed reduced infectivity over *wild type* ones (70% to 75% reduction over a single round infection assay, Figure 1C, left graph). To determine whether this defect could be observed using a WT HIV-1 clone (NL4-3, a widely used proviral clone), the same experimental system was used on viruses produced by transfection of HEK293T cells with NL4-3 and IFITMs. Upon exo-RT normalization, virions were used to challenge HeLaP4 cells and viral infectivity was measured by β-galactosidase assay (MAGI) 24 hours afterwards, taking advantage of the HIV-1-LTR-β-Gal reporter stably integrated in these cells (Figure 1C, right graph). Under these conditions, IFITMs imparted a similar infectivity defect to produced viruses (from 70 to 90% reduction for the different IFITMs).

### The effect of IFITMs on virion particles infectivity is exerted against different retroviruses and envelope pseudotypes

To determine the extent of this phenotype, IFITMs were tested according to the same experimental approach described above using: HIV-1 vectors pseudotyped with the Gibbon ape leukemia virus envelope (GALV) and with the feline leukemia virus RD114 envelope (RD-TR, containing the cytoplasmic tail of the MLV amphotropic envelope for efficient pseudotyping of lentiviral particles, as described in [29], Figure 1D), or vectors derived from different GFP-coding retroviruses (simian immunodeficiency virus, SIV_MAC_; murine leukemia virus, MLV) along with HIV-1, all pseudotyped with the pantropic Envelope VSVg (Figure 1E). After virus purification and normalization, viral particles were used to challenge HEK293T cells prior to flow cytometry analysis 3 days afterwards. Under these conditions, viruses produced in the presence of the different IFITMs displayed reduced infectivity that ranged from 90% in the case of SIV_MAC_-VSVg to 40% in the case of HIV-1-GALV or HIV-1-RD-TR. IFITM3 seemed to exert a lower effect on the infectivity of MLV-VSVg in comparison with IFITM1 and −2, yet this defect was clearly detectable and statistically significant. Overall, these results indicate that the expression of IFITMs during the phase of viral particles production impairs the infectivity of the retroviruses tested here.

### The infectivity defect of virions produced in the presence of IFITMs is observed in primary cell targets of HIV-1 infection

To determine whether the infectivity defect of HIV-1 viral particles produced in the presence of IFITMs was target cell type specific, virions produced as described above were used to challenge primary monocyte-derived dendritic cells (DCs), MDMs or activated PBLs using appropriate Env pseudotypes (Figure 1F). The infectivity of viral particles was then assessed by measuring the amount of GFP-positive cells 3 days afterwards. Even using different target cells, viruses produced in the presence of IFITMs displayed a characteristic decrease in infectivity, indicating that this phenomenon is independent from the target cells.

### IFITMs are incorporated in HIV-1 viral particles

**Figure 2:**
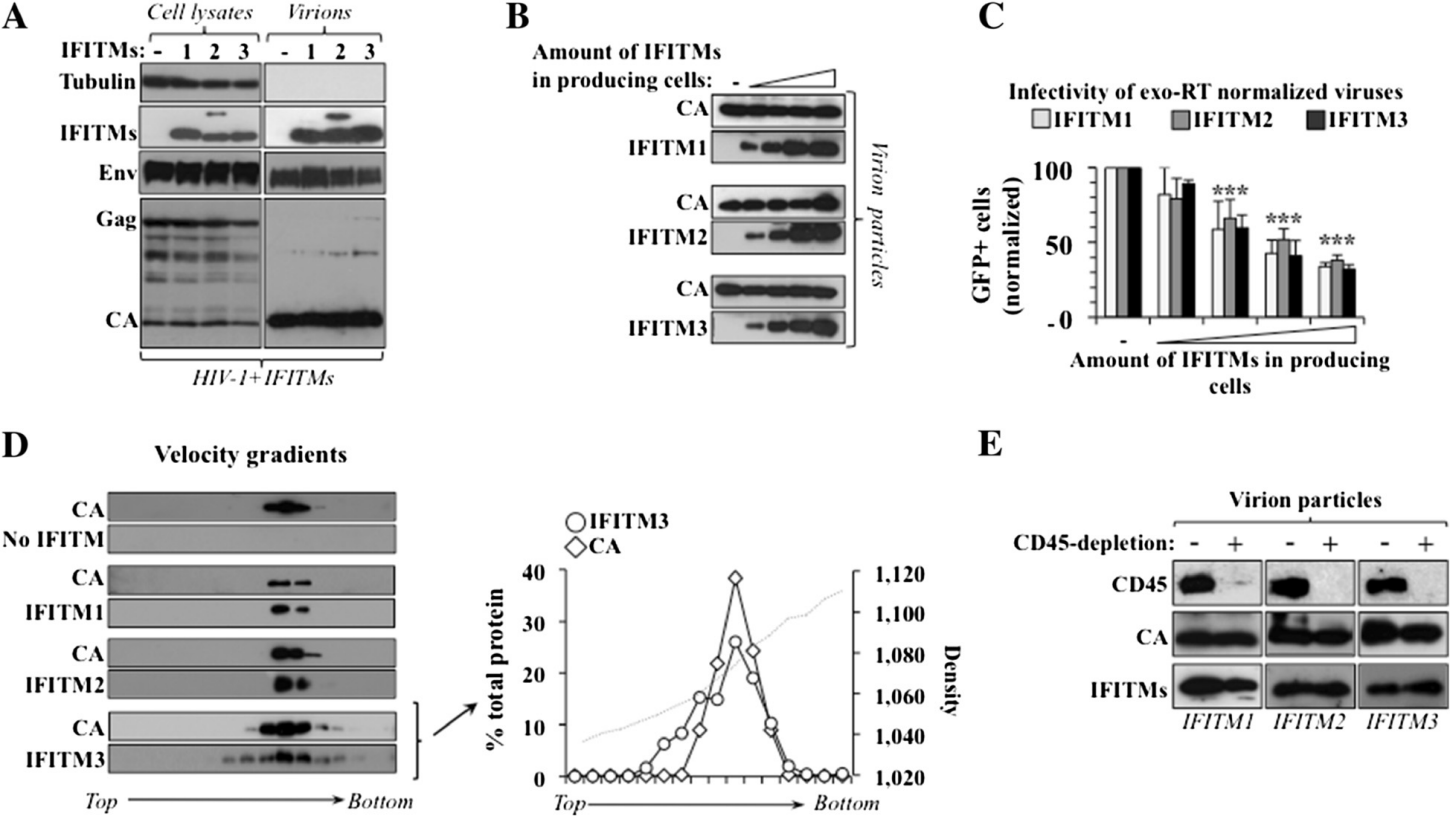
**IFITMs are HIV-1 virion associated proteins. A)** HEK293T cells were transfected with DNAs coding IFITMs and HIV-1 and two days after, both cell lysates and supernatant purified by ultracentrifugation through a 25% sucrose cushion were harvested and analyzed by WB. The panels present typical results obtained out of 10 independent experiments. **B** and **C)** HEK293T cells were transfected as above by maintaining a fixed amount of Gag-Pol/Env and by varying the amount of DNAs coding the different IFITMs. Virion particles were purified by ultracentrifugation, normalized by exo-RT and either analyzed by WB to determine the amount of IFITMs incorporated onto the virion particles **(B)**, or used to challenge HEK293T cells to determine their infectivity **(C)**. **D)** Virions produced in the presence of IFITMs were first concentrated and purified through sucrose as described above then layered onto a linear OptiprepTM velocity gradient (5 to 20% w/v) for an ultracentrifugation step of 45 minutes at 28,000 rpm. Aliquots were harvested from the top of the gradient, precipitated with TCA and analyzed by WB. The proportion of CA and IFITMs present in each fraction with respect to the total CA and IFITMs in all fractions was determined by densitometry and is presented here solely for IFITM3 due to space constraints. The density of each fraction was determined prior to TCA precipitation with a bench densitometer and is presented here as a dotted grey line (g/mL). The graph and associated WB panels are representative of 3 independent experiments. **E)** Virion particles produced from SupT1 cells stably expressing Flag-IFITMs were subjected or not to CD45 depletion, prior to WB analysis. The Western blot panels are representative of 3 independent experiments.

To determine how IFITMs affected HIV-1, cell lysates and viral preparations obtained after DNA transfection and virion purification by ultracentrifugation through 25% sucrose were analyzed by WB (Figure 2A). Under these conditions, cell lysates displayed a robust expression of all viral proteins tested. When viruses were analyzed, no notable variations were observed in the amount of Gag and Env present in the different preparations produced in the presence or absence of IFITMs (Figure 2A for a WB analysis and Additional file 1: Figure S1B for a precise quantification of gp120 and p24 by ELISA). Surprisingly, IFITMs were detected in viral preparations, suggesting that they could be virion-associated proteins. Next, we produced HIV-1 viral particles in the presence of a variable amount of IFITMs by transfecting different amounts of IFITM-coding DNAs for a constant amount of Gag-Pol and Env DNAs. Purified virion particles were then normalized by exo-RT and either analyzed by WB (Figure 2B), or used to challenge HeLaP4 cells (Figure 2C). IFITMs were incorporated in a dose dependent manner in virion particles and the infectivity of virion particles decreased proportionally to the levels of IFITMs incorporated, overall suggesting a dose response inhibition of IFITMs on viral infectivity.

To further support the finding that IFITMs are *bona fide* virion-associated proteins, supernatants obtained and purified after transfection of HEK293T cells with HIV-1 and IFITMs were layered onto a linear OptiprepTM gradient (5 to 20% w/v) and then ultracentrifuged for 45 minutes at 28,000 rpm for velocity gradient analyses. Aliquots were harvested from the top of the gradient, precipitated with TCA and analyzed by WB (Figure 2D). The signals obtained after WB were quantified by densitometry and were plotted here to indicate the percentage of each protein present in each fraction with respect to the overall protein present (Figure 2D, for space constraint the graph is presented only for IFITM3). Under these conditions, more than 95% of the total IFITMs co-migrated with HIV-1 CA in velocity gradients. These results along with similar co-migration observed between these proteins upon linear sucrose equilibrium density gradients (data not shown) indicate that IFITMs are virion-associated proteins.

Given that a recent report suggested that IFITM3 could be associated to exosomes upon overexpression in HEK293T cells [30] and given that exosomes share numerous characteristics with retroviral particles, we carried out CD45-depletion assays to determine whether IFITMs were associated to HIV-1 virion particles or to co-purifying exosomes. This assay takes advantage of a technique developed by the Ott lab that is based on the fact that CD45 is incorporated in exosomes, but is excluded from retroviral particles [31]. Given that CD45 is a T cell marker, we first obtained stable cell lines expressing each IFITM in SupT1 cells and then infected these cells with high MOIs of HIV-1 to obtain a large number of virion producing cells. Virion particles were then retrieved 6 days after and were then treated with CD45-magnetic beads or not prior to WB analysis (Figure 2E). Under these conditions, CD45 was efficiently removed by viral preparations. However, the amount of IFITMs present in HIV-1 viral preparations was unaffected by the treatment, except for a small decrease observed in the case of IFITM1. Overall, these results indicate that IFITMs are *bona fide* virion-associated proteins.

### IFITMs partly co-localize on intracellular membranes with HIV-1 Gag

**Figure 3:**
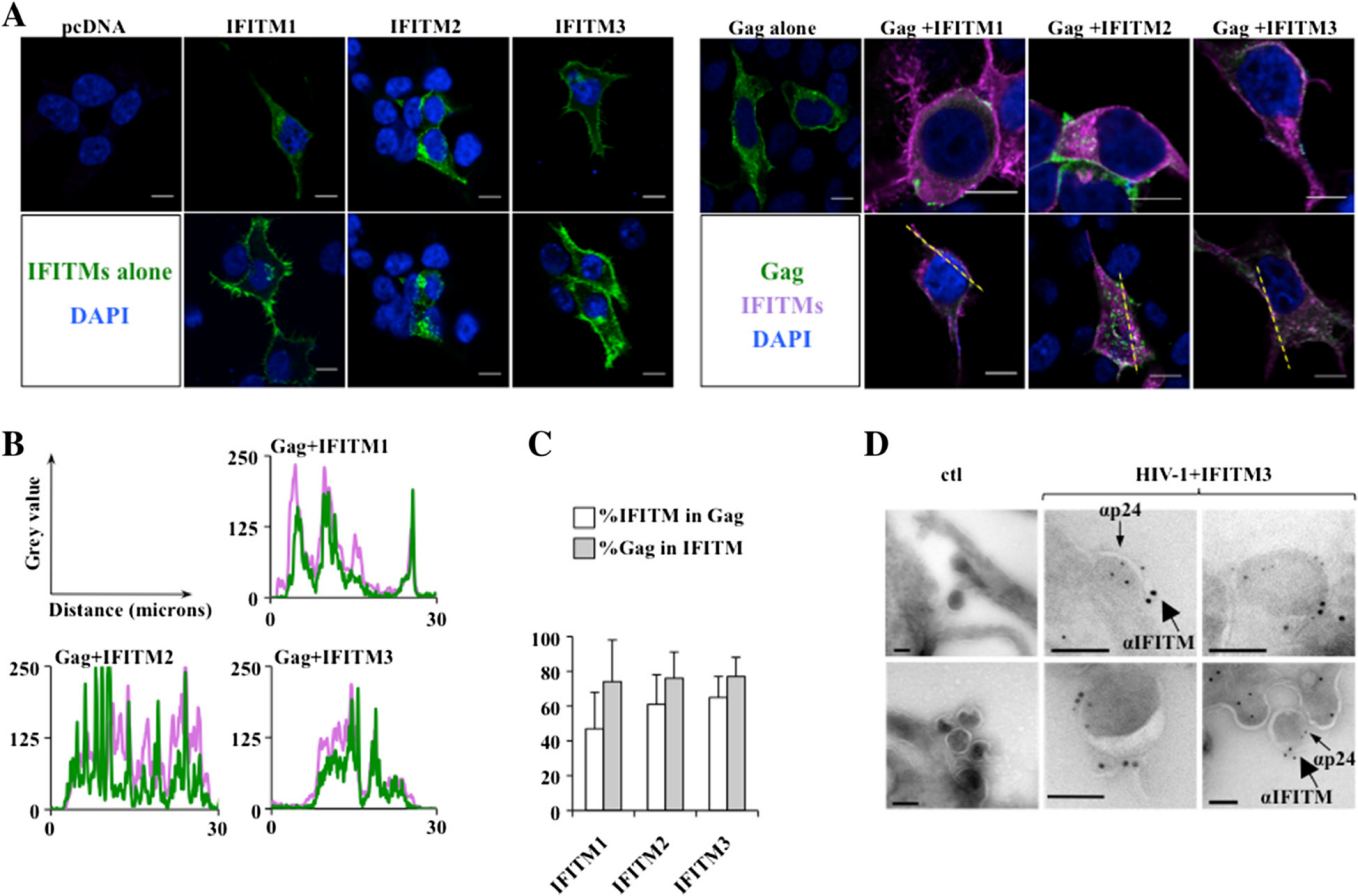
**IFITMs partly co-localize with HIV-1 Gag in virus-producing cells and coalesce with Gag into budding particles. A)** To determine the intracellular distribution of IFITMs and Gag, HEK293T cells were transfected as mentioned above with the addition of a small amount of an HIV-1 Gag-GFP fusion protein (1/10 of WT Gag-Pol). Cells were then fixed 24 hours after and analyzed by confocal microscopy. Representative panels of more than 100 cells per condition are shown here. Scale bars: 10 μm. **B)** Co-localization corresponding to the yellow lines of **(A)** was performed using the Plot Profile tool in Image-J. **C)** The extent of reciprocal co-localization of IFITMs and Gag was quantified using the Manders overlap coefficient (Fuji image software). **D)** HEK293T cells co-transfected with IFITM3 and HIV-1 were analyzed by immuno-gold labeling with anti-p24 and anti-Flag antibodies (5 and 10 nm beads, respectively, as indicated). Negative controls consisted of cells transfected with control DNA. Scale bars: 200 nm. All panels present typical results obtained out of 2 to 3 independent experiments.

To determine how IFITMs could be embarked into HIV-1 viral particles, we analyzed the degree of intracellular co-localization existing between IFITMs and Gag, the main structural element of retroviral particles. To this end, cells were transfected as above in the presence of a small amount of Gag-GFP, prior to confocal microscopy analysis (Figure 3A and B). As previously reported, all IFITMs were found at the plasma membrane, although more specific patterns could be observed for individual IFITMs (more prominent intracellular localization of IFITM2 and higher cell membrane distribution of IFITM1). HIV-1 Gag displayed an heterogeneous and mostly punctuate intracellular localization pattern, as extensively reported by others [32] and not surprisingly the two signals overlapped at least partially (Figure 3B, depicts a two-dimensional graph presenting pixel intensities for the cells presented above). The degree of reciprocal colocalization between Gag and IFITMs was more carefully quantified by measuring the Manders overlap coefficient (Figure 3C). From 50 to 65% of IFITMs was found to co-localize with Gag and about 70% of Gag co-localized with IFITMs, which is overall not surprising in light of the natural intracellular distribution of these proteins. No major relocalization of either IFITMs or Gag was noted upon co-expression, indicating that these proteins are unlikely to influence the trafficking of each other. So, overall this analysis indicates that in light of their natural membrane distribution, IFITMs can find themselves at sites in which HIV-1 virion particles assembly takes place, providing a reason for their incorporation in HIV-1 virions.

### IFITMs coalesce with HIV-1 budding virions

To further confirm that nascent HIV-1 viral particles could recruit IFITMs, cryo-EM was performed on cells co-expressing HIV-1 and IFITM3 using anti-p24 and anti-Flag antibodies conjugated to gold beads of different sizes (Figure 3D). The results obtained with this analysis indicated that IFITMs are indeed present at sites of Gag budding, further supporting the notion that IFITMs are truly incorporated into HIV-1 virion particles.

### Endogenous IFITMs are incorporated in HIV-1 particles produced from different cell types

**Figure 4:**
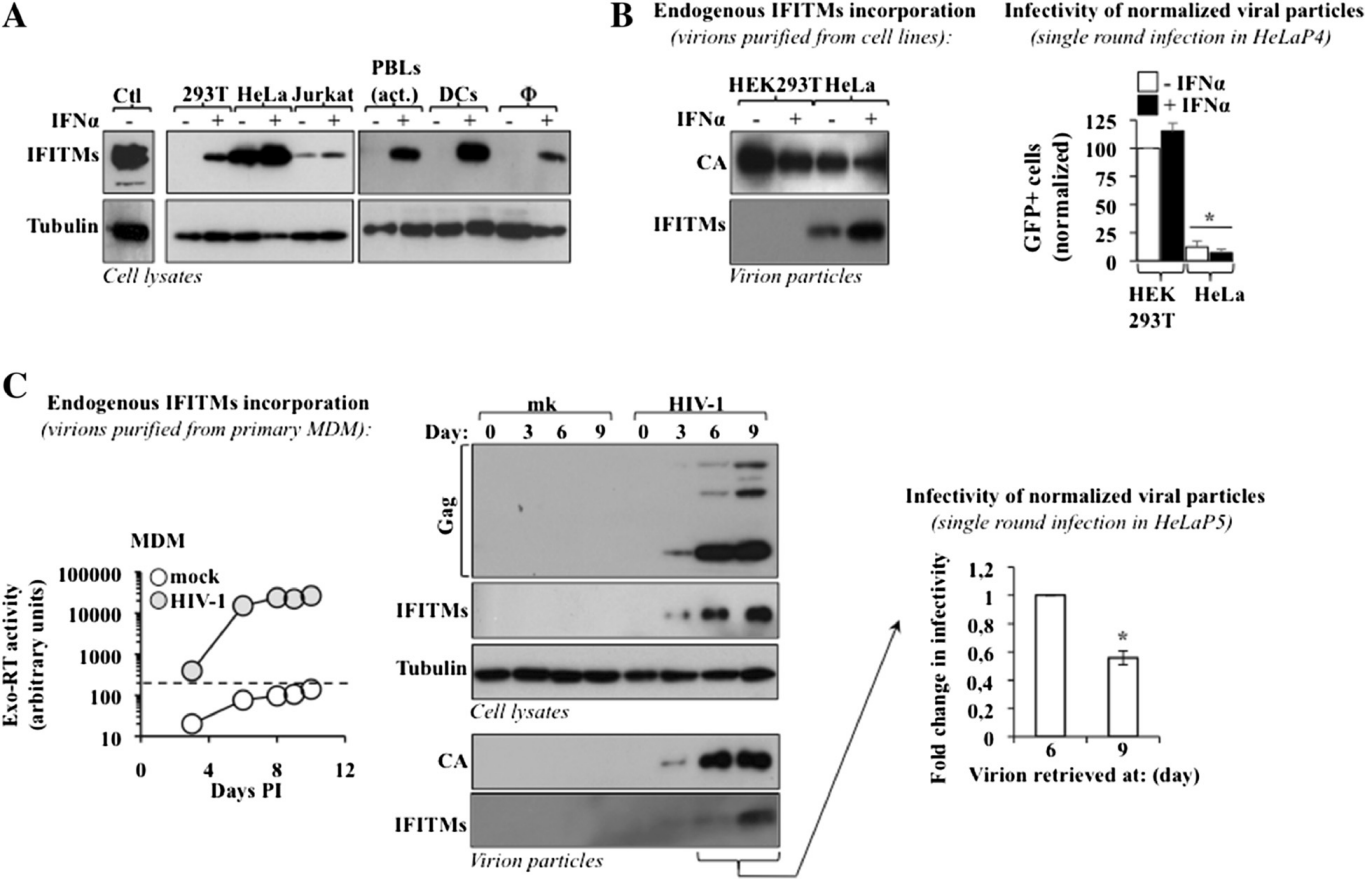
**IFITMs are interferon-regulated proteins that display heterogeneous cell type dependent expression and appear incorporated in HIV-1 virions proportionally to their intracellular levels. A)** The ectopic expression levels of IFITMs in HEK293T cells used before were compared to the endogenous expression of IFITMs in different cells, incubated or not with IFNα (at 1000 U/mL for 24 hours). Given that the antibodies in our hands did not distinguish between IFITM members anti-IFITM1, −2 and −3 antibodies were used together, so that all three forms are recognized. **B)** HEK293T and HeLaP4 cells were transfected with DNAs coding HIV-1-gfp and twenty-four hours later cells were stimulated with 1000U/mL of IFNα for further two days. Virions were then purified and analyzed by WB and upon exo-RT normalization they were used to challenge HeLa P4 cells. **C)** Primary macrophages obtained upon differentiation of monocytes in M-CSF for 4 days were challenged with an MOI of 0,1 of the R5-tropic HIV-1 strain ADA. Aliquots of the supernatant were harvested every few days and the extent of viral spread through the culture was measured by exo-RT activity. Cells and virion particles were harvested at the indicated time points after infection. Virion particles obtained from day 6 and 9 were normalized by exo-RT activity, while the amount of virus obtained at day 3 was too low to be quantified further. Exo-RT normalized virions obtained at day 6 and 9 were used to challenge HeLaP5 cells for a MAGI assay. The graph presents a typical replication curve obtained upon infection of ADA in primary MDM and the dotted line represents the limit of detection of the assay. All WB panels present representative results obtained out of 3 to 5 independent experiments and donors, while the graphs present averages and SEM of 3 independent experiments.

To determine whether the incorporation of IFITM proteins onto HIV-1 virions could be observed in conditions of endogenous expression, we first tested several antibodies for their ability to specifically recognize individual IFITM members (on HEK293T cells transfected with the individual IFITMs, Additional file 2: Figure S2). This analysis indicated a certain level of cross-recognition between antibodies (particularly with the anti-IFITM2 and −3 antibodies). For this reason, we decided to detect the endogenous expression of IFITMs using a pool of the 3 antibodies. We first determined the pattern of expression of IFITMs in different established cell lines and primary cells (Figure 4A). Given that IFITMs are interferon-stimulated genes, these cells were also stimulated for 24 hours with 1000 U/mL of IFNα. The basal expression of IFITMs varied among cell types and was undetectable in HEK293T cells, unless IFNα was provided. HeLa cells expressed instead robust levels of IFITMs even in the absence of IFNα, although this stimulus further increased their expression. Similarly, the primary human cells examined here expressed undetectable/low amounts of IFITMs under standard conditions, but expression of IFITMs was robustly induced by IFNα stimulation. This trend was reproducibly observed in four distinct donors, although the basal levels of expression of IFITMs displayed clear donor-to donor variations.

To determine whether endogenous IFITMs could be incorporated into HIV-1 particles, we first compared virions issued from HEK293T versus HeLa cells treated or not with IFNα (Figure 4B). Twenty-four hours after transfection with HIV-1 coding DNAs, cells were washed and then treated with 1000U/mL of IFNα for further 48 hours, prior to virion particle purification. Virion particles were then either analyzed by WB or used to challenge HeLaP4 cells upon exo-RT purification to determine their infectivity by flow cytometry analysis 3 days afterwards. When HIV-1 virions were produced in HEK293T cells, no detectable amounts of IFITMs were found, irrespectively of IFN stimulation suggesting that IFITMs may have to reach a certain amount to be promptly incorporated into virions. In contrast, virion produced in HeLa cells incorporated readily detectable levels of IFITMs and this incorporation was increased upon IFN stimulation. When virion particles produced in these conditions were normalized and used to challenge HeLaP4 cells, a drastic decrease in infectivity was observed in virions produced in HeLa cells versus viruses produced in HEK293T cells and IFN stimulation did not grossly modify these differences.

Next, a similar analysis was conducted on primary macrophages undergoing spreading HIV-1 infection. MDM were infected with replication competent HIV-1 (ADA at a multiplicity of infection, MOI, of 0,1). Cells were washed and aliquots of the supernatant were harvested at different days post infection to monitor viral spread by exogenous-RT activity (Figure 4C) and cell lysates and virion particles were also harvested at the indicated times post infection. The intracellular levels of IFITMs increased over time during spreading HIV-1 infection, in agreement with the upregulation of an IFN-dependent transcriptional program described in a number of previous studies ([33-36]), although not all ([37]). As expected, when virion particles produced at different days after infection were examined, IFITMs were incorporated into HIV-1 particles proportionally to their intracellular levels of expression. Of note, only a limited virus production was observed at day 3 post infection, as expected from the low levels of replication ongoing at this early time point. Virus production was however more robust at later time points, so that the intrinsic infectivity of virions produced from MDM at day 6 and 9 could be assessed after a MAGI assay on HeLaP5 cells. Under these conditions, virions obtained at day 6 displayed higher infectivity than viruses obtained at day 9, in agreement with the higher incorporation of IFITMs in the latter.

Overall, this set of data indicates that endogenous IFITMs are incorporated into virion particles and exert an antiviral effect in a manner that seems proportional to their intracellular levels. Given that viral infectivity is likely multifactorial, we believe this data suggest that IFITMs may be an important parameter of viral infectivity, although we believe it unlikely to be the only one.

### Downregulation of all 3 IFITMs increases the infectivity of HIV-1 viral particles

**Figure 5:**
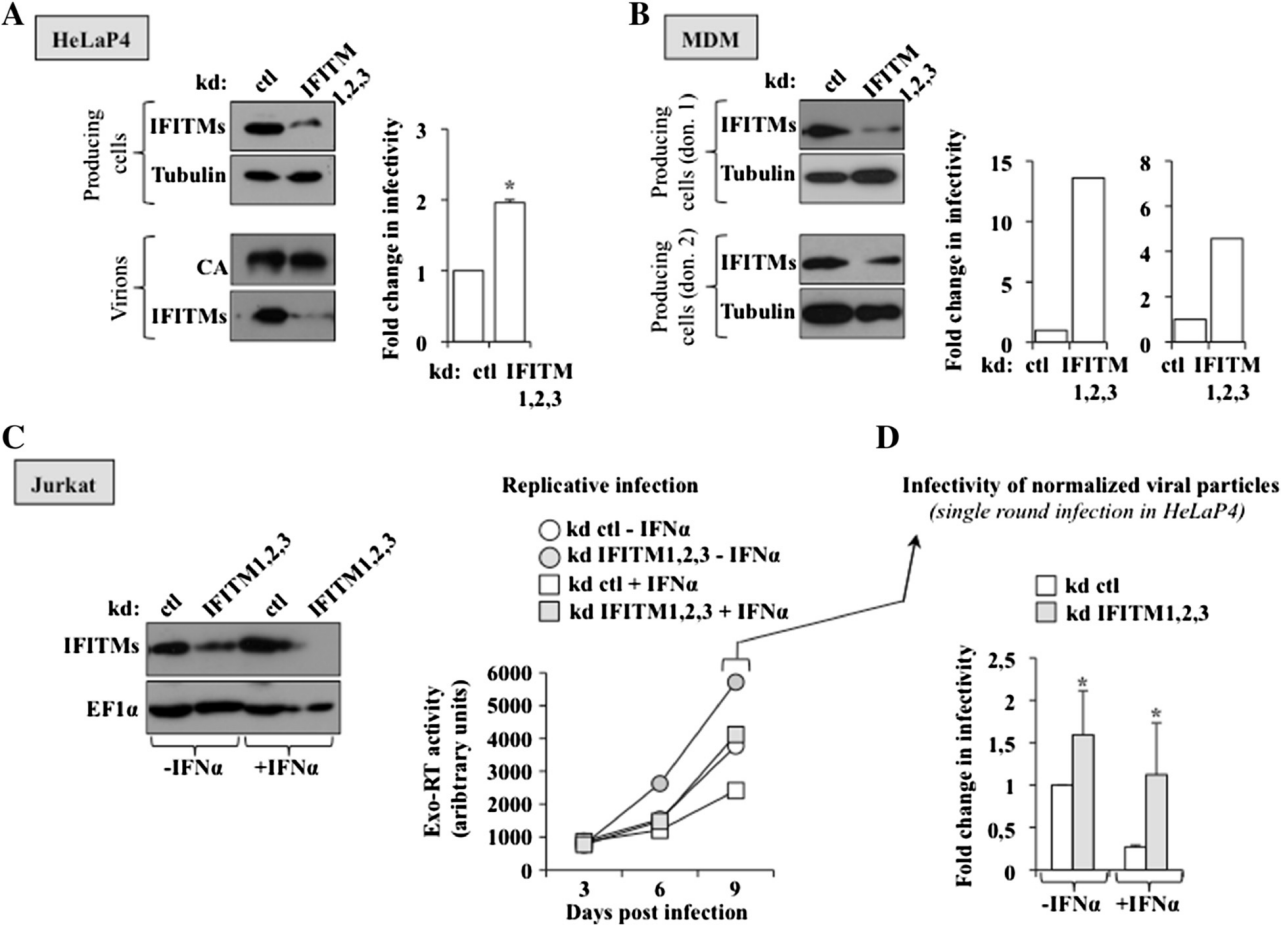
**IFITMs silencing in HIV-1 producing cells results in virions of increased infectivity.** HIV-1 vectors coding for control (Luciferase) or IFITMs specific target sequences were obtained by DNA transfection of HEK293T cells, normalized and used on target cells. **A)** HeLaP4 were shortly selected with Puromycin (present in the vector) and then challenged with an MOI of 1 of replication competent HIV-1 virus (NL4-3) to obtain a large proportion of virus producing cells. After cell washing and trypsin treatment, newly produced viruses were recovered 2–3 days afterwards and virions and cell lysates were examined by WB at this time. The infectivity of exo-RT normalized virions was determined on HeLaP4 cells by MAGI assay. **B)** To improve silencing efficiency in MDM, vectors were used along with an MOI-equivalent of 0,5 of VLPs-Vpx. Three days after, MDM were challenged with an MOI of 1 of replication competent ADA virus, prior to extensive cell washing and trypsin treatment. Newly produced viral particles were retrieved 4 to 6 days after and their infectivity determined after exo-RT normalization on HeLaP5 cells. **C)** Knockdown Jurkat cells were obtained as in A. Cells were then challenged with an MOI of 0,1 of replication competent NL4-3 and spreading infections were analyzed by harvesting aliquots of the culture supernatant at different times post infection. Viral spread was determined by exo-RT activity. **D)** The intrinsic infectivity of viral particles was determined as above on the supernatants of cells obtained 9 days post infection. WB panels present knockdowns obtained for the different cell types and the graph presents averages and SEM obtained in 3 to 4 independent experiments and donors. *; statistically significant difference after a Student *t* test: p ≤ 0,05. The replication curve shown in C depicts a typical result obtained out of 3.

To further support the argument that IFITMs affect the infectivity of newly produced viral particles under endogenous conditions, we silenced IFITMs from virus-producing cells and given that each IFITM exerts an antiviral effect, the three of them were targeted simultaneously. We first used HeLaP4 cells that express IFITMs at steady state. HeLa cells were challenged with miR30-shRNAs specific for the 3 IFITMs or control target sequences (*luciferase*), and shortly selected with puromycin. Then, cells were challenged with replication competent HIV-1 (NL4-3 at an MOI 1) to obtain a consistent amount of virus-producing cells. After extensive cell washing and trypsin treatment to remove non internalized virus, cells were re-seeded and two days after, cells were lysed and newly produced viral particles were purified, normalized by exo-RT and used for a WB analysis, as well as to challenge target cells (Figure 5A). As expected, downregulation of the intracellular levels of IFITMs led to viruses that incorporated less IFITMs. When the infectivity of exo-RT normalized viral particles produced from control versus IFITM knockdown cells was assessed, the formers displayed a statistically significant increase in infectivity (two fold).

Next, we sought to achieve the same goal in primary MDM obtained from 2 donors. Silenced MDM could not be maintained long enough to perform a classical spreading assay, nor sufficient virus could be obtained for a WB analysis of viral particles. However, this setup was sufficient to obtain viral particles whose infectivity could be analyzed by MAGI assay.

To increase silencing efficiency in these cells, lentiviruses coding control or IFITM-specific miR30-shRNAs were provided along with virion-like particles containing the Vpx protein of SIV_MAC_ (VLPs-Vpx), tool that allows an efficient step of reverse transcription and infection of lentiviruses in myeloid cells [38,39]. Two days afterwards, cells were challenged as above with R5-tropic replication-competent ADA at an MOI of 1 prior to extensive cell washing and trypsin treatment. Viral particles produced by knockdown cells were then harvested 4 to 6 days after and upon exo-RT normalization, viruses were used on HeLaP5 cells (expressing the CCR5 co-receptor) for a MAGI assay (Figure 5B). Under these conditions, viruses produced in MDM silenced for IFITMs displayed a remarkable increase in their infectivity over a single round infection assay, confirming the positive effect that the removal of IFITMs plays on the infectivity of viral particles. Of note, the basal expression of IFITMs was higher in miR30-shRNA transduced MDM, irrespectively of the target sequence, when compared to untreated cells (for example, compare the basal levels of expression observed here with the one of Figure 4A). We believe this is due to the detection of the HIV vectors used for silencing, or to the detection of its end products, ie double stranded RNAs, as described in [40-42]. However, since equal conditions were used for control and specific knockdowns, we believe this not to be a confounding factor in the analysis of the results.

Lastly, we silenced IFITMs in Jurkat cells and since IFITMs were moderately upregulated upon IFNα treatment, we compared the effects that IFNα played on viral replication in both control or IFITM-knockdown cells (Figure 5C). Silenced cells were challenged with replication competent NL4-3 and viral replication was monitored through the accumulation of exo-RT in the culture supernatants at different days post infection. Under these conditions, viral replication was increased by IFITMs silencing in both IFN-stimulated and unstimulated conditions over control silenced cells, indicating that IFITMs may provide already a basal level of resistance even in non stimulated Jurkat T cells. Given that in addition to the direct decrease in viral particles infectivity that we describe here, IFITMs have been previously described to affect the entry of HIV-1 when expressed in target cells [9] and given that the assay of spreading infection does not allow the distinction between these two effects, viral particles retrieved at the end of the culture were normalized by exo-RT activity and used to challenge HeLaP4 cells to determine their intrinsic infectivity in a single round infection assay (Figure 5D). Under these conditions, the infectivity of viral particles produced in IFITM-knockdown cells was higher than the one of viruses produced in control cells and this increase was of 1,6 fold in unstimulated Jurkat cells, but reached 5 fold in IFN-stimulated cells, suggesting again that IFITMs do play an antiviral role in Jurkat cells that is exacerbated upon IFN stimulation. Of note, we have already determined that IFNα does not influence the early phases of infection in HeLa cells [43], so that its presence is unlikely to be a confounding factor in the determination of the infectivity of viral particles in this cell type. Overall, these results further strengthen the notion that IFITMs negatively interfere with the infectivity of HIV-1 viral particles and do so in different cell types in which they are naturally expressed.

### HIV-1 particles incorporating IFITMs display an entry defect in target cells

**Figure 6:**
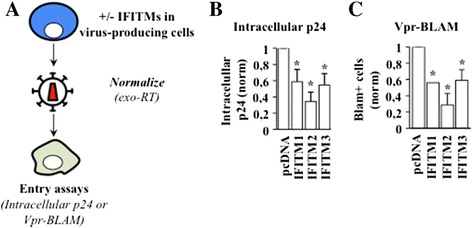
**IFITMs incorporation into HIV-1 viral particles affects viral infectivity by interfering with entry of the virus in target cells.** Normalized NL4-3 Env bearing HIV-1 virions produced in the presence or absence of IFITMs were bound to HeLaP4 cells at 4°C prior to extensive cell washing. The temperature was then raised at 37°C to induce entry. The extent of entry was then measured according to the two assays schematically depicted in **A**. **B)** Two hours after entry, cells were extensively washed, treated with trypsin to remove external virion particles and then lysed. The amount of cell-associated p24 was measured by ELISA. **C)** After exo-RT normalization, equal amounts of viruses incorporating Vpr-BLAM were used to challenge HeLaP4 cells for 2 hours. Cells were then incubated with the fluorescent dye CCF2 prior to FACS analysis. Graphs present averages obtained from 3 to 8 independent experiments. *p ≤ 0,05, according to a Student *t* test.

To determine at which step the incorporation of IFITMs affected viral infectivity, virion particles produced in presence or absence of IFITMs were purified and normalized as described above and used to challenge target cells according to two methods (schematically indicated in Figure 6A). Target cells were incubated with an equal amount of viral particles at 4°C, extensively washed and then shifted at 37°C to induce virus entry into the cell. After 2 hours, cells were treated with trypsin to remove extracellular virus and the amount of intracellular p24 was determined by ELISA. Under these conditions, all IFITMs-HIV-1 particles presented an entry defect, indicating that IFITMs interfered with the ability of virion particles to enter target cells (from 40% to 70% Figure 6B). To further support this argument, viruses were produced in the presence of Vpr-Blam. This protein is incorporated into virion particles and is released in the cytoplasm of target cells after viral-to-cell membrane fusion where it can cleave a fluorescent dye [44]. When viral particles incorporating Vpr-Blam were analyzed a similar decrease in entry was measured for virions incorporating IFITMs as compared to WT (Figure 6C, from 50 to 70%). Although this defect appeared more pronounced in the case of IFITM2 than of IFITM1/3, this difference did not reach statistical significance.

Overall, these results indicate that the incorporation of IFITMs onto HIV-1 viruses interferes with the ability of viral particles to enter in target cells.

## Discussion

In the work presented here we describe a novel feature related to the biology of IFITMs, namely their ability to be incorporated into HIV-1 virion particles and to decrease the particle infectivity. So far, IFITMs have been essentially studied in the context of target cells, where their overexpression induces a strong antiviral phenotype by trapping incoming viral particles in endosomes. This antiviral activity is broad and targets, albeit with different efficiency, a large panel of viruses including HIV-1 [3-12]. Here, we report that the presence of IFITMs in virus producing cells leads to the production of virions of decreased infectivity. In light of the largely membrane distribution of the different IFITMs their incorporation into retroviral particles is not surprising. The diverse spectrum of envelopes and of retroviruses on which IFITMs exert similar effects leads us the hypothesize that this mechanism of inhibition does not target a specific viral domain, but rather takes advantage of the manner in which HIV and more generally retroviruses assemble. In this respect, the membrane distribution of IFITMs strongly suggests that passive incorporation is the most plausible explanation for their packaging in retroviral particles. This incorporation does not seem to occur to the detriment of the one of Env, as IFITMs do not modify the Env to Gag ratio of virion particles (data not shown).

At present, although our preferred hypothesis is that the physical presence of IFITMs is required to lower the infectivity of viral particles, we cannot exclude the possibility that IFITMs act on producing cells and that this action in turn leads to the production of viral particles of decreased infectivity. Despite the fact that the overall restrictive phenotype would nonetheless remain, the underlaying mechanism would be profoundly different, as in this case the incorporation of IFITMs would be probably a mark, but not a cause of the infectivity defect described here. We believe the future identification of IFITM mutants that have lost the ability to be incorporated into viral particles may help us distinguish between these possibilities and efforts toward this goal are ongoing.

IFITMs-HIV-1 particles are impaired at entry, the same step inhibited when IFITMs are present in the opposite topology, i.e. in target cell membranes [17,26]. At present, the mechanism through which IFITMs block endosomal fusion of incoming viruses remains controversial [25,26] and this mechanism could be molecularly distinct from the one at play here. Multiple hypotheses have been put forward to explain the antiviral effect of IFITMs in target cells. IFITMs have been reported to increase the intracellular levels of cholesterol [25] and variations in cholesterol have been associated to defects in membrane fusion, viral production and are likely to play an important role in multiple physiological processes [45-48].

Alternatively, IFITMs have been proposed to sterically rigidify membranes in which they insert leading to fusion inhibition, as recently proposed [26]. This hypothesis is supported by the finding that IFITMs can interact between themselves, although the extent of this multimerization has not been fully examined [49]. Whether these mechanisms are at play here and more importantly whether the same mechanism/s of inhibition is at play in target cells and in virion particles remains to be determined.

When compared to their effect on other viruses, as for example Influenza virus, IFITMs display a milder antiviral phenotype against HIV-1 *ex vivo*. However, this does not preclude an important role of IFITMs in shaping HIV-1 quasi-species evolution *in vivo*, possibly as part of the more complex interferon response. An increasing number of reports indicate that the susceptibility of HIV-1 to IFNα changes during the course of the disease in infected patients [50,51] and HIV-1 strains displaying increased resistance to IFITMs have been selected *ex vivo* [52]. These results suggest that the evolutionary pressure exerted by IFITMs on HIV-1 is likely not neutral. In this respect, it will be of interest to determine whether multiple primary HIV-1 strains display distinct susceptibilities to IFITMs and whether particular IFITM haplotypes can be associated to distinct HIV-1 outcomes, as is the case for Influenza virus [13].

## Conclusions

In conclusion, our study uncovers a novel interesting aspect of the biology of IFITMs and provides an interesting example of how a single restriction factor may interfere at two different steps of the viral life cycle using a seemingly similar mechanism. These findings may apply to other pathogens and yield a more complex view of the manner in which this emerging family of restriction factors can interfere with viral pathogens.

## Methods

### Plasmids and reagents

N-term Flag-IFITMs-DNAs were obtained from Dr. Guo (Drexel University, Doylestown, USA). The following DNA expression constructs have been described before: Gag-Pol + non-structural proteins and corresponding viral genomes coding GFP that yield single-cycle infection-competent viruses derived from different retroviruses [38]; replication-competent HIV-1 proviral clones (X4- or R5-tropic, NL4-3 and ADA, respectively); VSVg and HIV-1 envelopes; the Vpr-Blam and Gag-GFP [32,44]; GALV and RD-TR [29]. For WB and immuno-gold analyses, the following antibodies were used: anti-Tubulin (Sigma), anti-Flag (F7425, Sigma), anti-Gag/p24 (clone 183-H5C from the AIDS Reagents Program of the NIH), anti-Env (for WB: #ab21179, Abcam), anti-IFITM1, −2 and −3 (#60074-1-Ig, 12769-1-AP and 11714-1-AP, respectively, Proteintech), anti-CD45 (Becton Dickinson). IFNα (Eurobio) was used at a final concentration of 1,000 U/mL.

### Viral production, titration and infection

Viral particles were produced by calcium phosphate DNA transfection of HEK293T cells (obtained through the CelluloNet facility of the UMS3444 Biosciences Gerland). Single-cycle infection-competent viruses were produced by co-transfection of 3 DNAs coding Gag-Pol, a miniviral genome coding GFP and Env (for a 10 cm plate: 4, 4 and 1 μg each, respectively) and IFITMs were added at a Gag-Pol/IFITMs ratio of 1 to 3. When indicated, this ratio was lowered to 1 to 0,07 by modifying the amount of IFITM transfected for a constant amount of Gag-Pol and Env coding DNAs. Media was replaced 12 hours after transfection and viral supernatants were collected 48 hours after. Supernatants were first centrifuged at 2,000 rpm for 10 minutes, then filtered through a 0,45 μm syringe-filter, prior to purification by ultracentrifugation at 25,000 rpm for 2 hours through a 25% (w/v) sucrose cushion. After ultracentrifugation, the pellet was resuspended in DMEM and normalized by either exogenous-RT activity or by an in-house anti-p24 ELISA, as described [39]. Viral infectivity was determined 3 days after cell challenge by flow cytometry analysis (in the case of GFP-coding vectors) or 24 hours PI by β-gal assay in HeLaP4 cells (stably expressing the CD4/CXCR4 receptors plus an HIV-1-LTR-β-gal reporter cassette, obtained through the CelluloNet facility of the UMS3444 Biosciences Gerland) with replication-competent HIV-1. The Env-Gag ratio of viral particles produced in the presence or absence of IFITMs was carried out by gp120 and p24 ELISA, as described [53].

### CD45-depletion assays

This procedure was essentially described in [31]. To obtain virions issued from CD45 expressing cells (CD45 is indeed mostly a T cell marker), SupT1 stably expressing Flag-IFITMs were challenged with replication-competent NL4-3 at an MOI of 3 to induce a rapid burst of infection and robust production of virion particles. Virions produced upon ongoing infection were harvested 6 days after infection and were incubated with magnetic beads coupled with an anti-CD45 antibody (Miltenyi) for 2 hours. Beads were then recuperated on a magnetic support following the manufacturer’s instructions. Bound and unbound material was analyzed by WB.

### Primary cells

Monocytes and PBLs were purified from the blood of healthy donors by successive Ficoll and Percoll gradients followed by negative depletion (Miltenyi and [39]. Monocytes were differentiated into macrophages (MDM) or DCs with M-CSF or GM-CSF/IL4 for 4 to 6 days (AbCys), while PBLs were activated with IL2 (150 U/mL, AIDS Reagents and Reference Program of the NIH) and PHA (1 μg/mL, Sigma). For replicative infections, cells were challenged with an MOI of 0,1 of ADA. Every 3–4 days aliquots of the cell supernatant were harvested and replaced with fresh media. The extent of viral replication in the cell culture was measured by exo-RT activity.

### Velocity gradients

Supernatants produced by transient DNA transfection of HEK293T cells were first concentrated by ultracentrifugation through a 25% sucrose cushion, as described in the Methods section, then resuspended and layered onto a linear OptiprepTM gradient (5 to 20% w/v) prior to ultracentrifugation for 45 minutes at 28,000 rpm. Fractions collected from the top of the gradient were then precipitated with a final concentration of 10% TCA and analyzed by WB. The intensity of the retrieved bands was quantified by densitometry. The amount of CA and IFITMs present in the different fractions was then normalized to the total amount of CA and IFITMs present in all fractions (set to 100%). Prior to precipitation the density of each fraction was measured using a bench densitometer.

### Confocal microscopy analysis

HEK293T cells were grown on 0,01% poly-L-lysine coated coverslips and analyzed 24 hours post-transfection with DNAs coding: IFITMs, HIV-1 Gag-Pol plus non-structural viral proteins, NL4-3 Env, a miniviral genome coding CD8, as well as a small amount of Gag-GFP (ratio 3:1:1:1:0,1). After Formalin fixation, cells were incubated with the following antibodies: anti-Flag (F7425, Sigma), followed by DyLight 649- conjugated sheep, or FITC-conjugated goat anti-rabbit IgG (STAR36D649 AbD serotec and FI-1000 Vector). DAPI-containing mounting medium was finally used (DAPI Fluormount G, Southern biotech). Images were acquired using a spectral Leica sp5 and analyzed with the Fiji software [54]. Two-dimensional graphs representing pixel intensities (gray level) were plotted along a 30-μm lines (yellow on Figure 3B), using Plot Profile tool in Image-J. The extent of reciprocal co-localization between Gag and IFITMs was quantified using the Manders overlap coefficient (Fuji image software) on more than 40 cells per condition.

### Ultra-thin cryosections and immunogold labeling

HEK293T cells transfected as described above were fixed for one hour with 4% paraformaldehyde in phosphate buffer (pH 7.6), washed and then infused with sucrose 2.3 M for 2 hours (4°C). Ninety nm ultra-thin cryosections were made at −110°C on a LEICA UCT cryoultramicrotome. Sections were retrieved with a methylcellulose 2%/sucrose 2.3 M mixture (1:1) and collected onto formvar/carbon coated nickel grids. Sections were incubated with anti-Flag and anti-p24 antibodies (F7425, Sigma and KAL-1, DAKO). After extensive washing, grids were incubated with gold-conjugated goat-anti-rabbit IgG (Aurion) and goat-anti-mouse IgG (10 and 5 nm, Sigma). Grids were washed, post-fixed in 1% glutaraldehyde and rinsed. Contrasting step was performed by incubating grids on drops of uranyl acetate 4%/methycellulose 2% mixture (1:10). The sections were imaged on a transmission electron microscope at 100 kV (JEOL 1011, Tokyo, Japan).

### Viral entry assays

Exo-RT-normalized viral preparations were used to infect HeLaP4 cells for 30 min at 4°C. Cells were then extensively washed and then shifted at 37°C for 2 hrs. Prior to lysis and p24 ELISA, cells were treated with trypsin to remove non-internalized virus. The Vpr-Blam assay was carried out according to a well-described protocol [44].

### Silencing experiments

miR30-shRNAs were introduced into the desired cell types by HIV-1 vector-mediated transduction [55]. Briefly, self-inactivating (SIN) lentivectors were produced by cotransfection of HEK293T cells using a viral genome bearing a miR30-shRNAs-Puromycin cassette (a mixture of two target sequences per gene was used). Virions were then purified and normalized by exo-RT, so that an identical viral input was used to challenge target cells with control or with the overall pool of IFITM1,2,3 specific miR30-shRNAs. The silencing procedure was then adapted experimentally for each cell type to reach the best possible compromise between high silencing efficiency and optimal cell survival. In the case of HeLaP4 cells, after a short Puromycin selection (4 days), cells were seeded and used as virus producing cells. To this end, cells were challenged with an MOI of 1 of replication competent HIV-1 (NL4-3) to obtain a substantial fraction of virus-producing cells. After extensive cell washing and trypsin treatment to remove non-internalized virus, cells were seeded and newly produced viruses were harvested 2 to 3 days afterwards from the supernatant of knockdown cells. Virions were normalized by exo-RT and used to challenge naïve HeLaP4 cells. The amount of infectious viral particles present in the different preparations was then assessed 24 hours later following a MAGI assay on these cells. For silencing experiments in primary macrophages, cells were similarly transduced in the presence of virion-like particles containing Vpx (VLPs-Vpx provided at an MOI-equivalent of 0,5) that increase the overall transduction efficiency of lentiviruses in human myeloid cells by removing a restriction at reverse transcription, according to a well-established protocol [39]. In this case, silenced cells were used in the absence of Puromycin selection and were challenged with an MOI of 1 of the R5 tropic replication-competent ADA. After extensive cell washing and trypsin treatment, virions were harvested 4 to 6 days afterwards and similarly normalized by exo-RT prior to challenge of HeLaP5 cells (expressing the CCR5 co-receptor) and MAGI assay. In the case of Jurkat cells, silenced cells were kept in Puromycin selection and used in replicative infections using the NL4-3 virus. Aliquots of the cell supernatant were harvested at different time points and the extent of viral spread was assessed by exo-RT activity. To determine the intrinsic infectivity of viral particles produced in Jurkat cells treated as mentioned in the text, viral particles were retrieved at day 9 post infection to obtain sufficient amount of virus for our analysis, then viral preparations were normalized by exo-RT activity and used to challenge naïve HeLaP4 reporter cells. The infectivity of normalized viral particles was then determined by a MAGI assay 24 hours later.

Target sequences were as follows: luciferase (acc.n° DQ188838): AGCTCCCGTGAATTGGAATCC; IFITM1 (acc.n° NM_003641.3): ATCTGTGACAGTCTACCATATT and CCCATATTATGTTACAGATAAT; IFITM2 (acc.n° NM_006435.2): ACCAGCCTCCCAACTACGAGAT and ACCCGATGTCCACCGTGATCCA; IFITM3 (acc.n° NM_021034.2): ACCCGACGTCCACCGTGATCCA and ACCCCCAACTATGAGATGCTCA.

## Additional files

Additional file 1: Figure S1. Expression of IFITMs does not grossly affects viral production and does not modify the Env to Gag ratio of viral particles. A) The overall amount of viral particles produced upon co-transfection of HEK293T cells with DNAs coding HIV-1 vectors and IFITMs was quantified by exo-RT activity. B) The amount of Env and of CA present in exo-RT normalized viruses obtained in the presence or absence of IFITMs was determined by ELISA and is presented here as a gp120/p24 ratio. The graphs present averages and SEM obtained with 3 to 8 independent experiments. *p ≤ 0,05, according to a Student *t* test. Additional file 2: Figure S2. Extent of cross-reactivity of commercially available anti-IFITM antibodies. To assess the specificity of commercial antibodies in our hands for the different IFITMs, HEK293T cells were transfected with each IFITM and cell lysates were then analyzed by WB.
