# Supplementary figures and images for: IFITM proteins are incorporated onto HIV-1 virion particles and negatively imprint their infectivity
Source: Retrovirology. 2014 Nov 25;11:103. doi: 10.1186/s12977-014-0103-y (PMC4251951; doi:10.1186/s12977-014-0103-y)

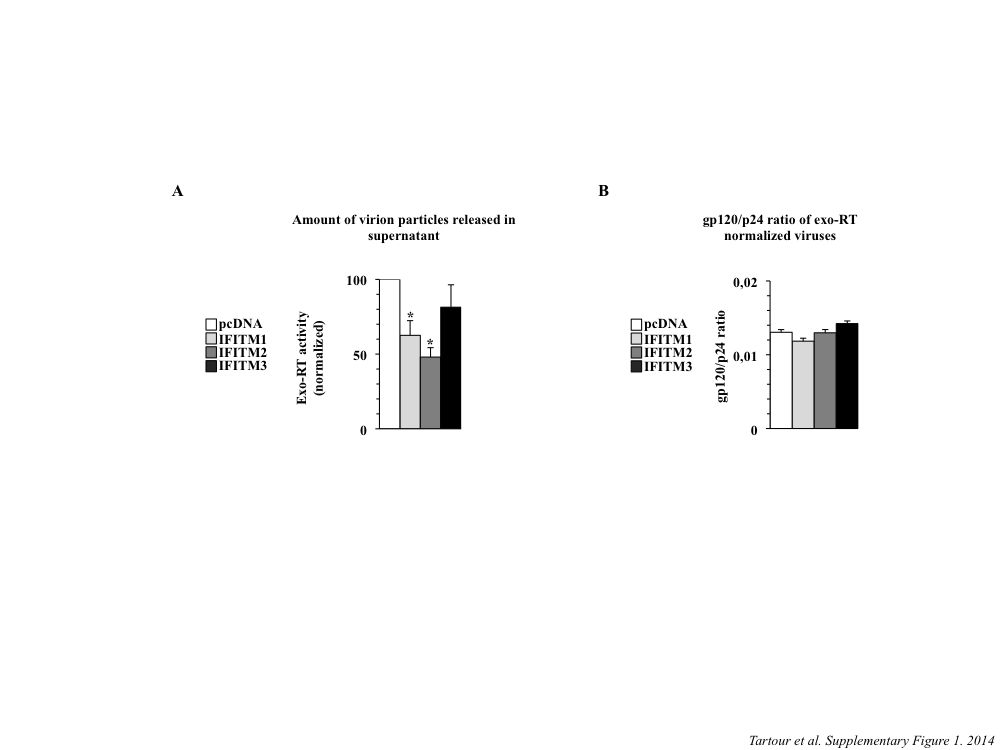

Supplement: Additional file 1: Figure S1. — Expression of IFITMs does not grossly affects viral production and does not modify the Env to Gag ratio of viral particles. A) The overall amount of viral particles produced upon co-transfection of HEK293T cells with DNAs coding HIV-1 vectors and IFITMs was quantified by exo-RT activity. B) The amount of Env and of CA present in exo-RT normalized viruses obtained in the presence or absence of IFITMs was determined by ELISA and is presented here as a gp120/p24 ratio. The graphs present averages and SEM obtained with 3 to 8 independent experiments. *p ≤ 0,05, according to a Student t test. [file 12977_2014_103_MOESM1_ESM.tiff]

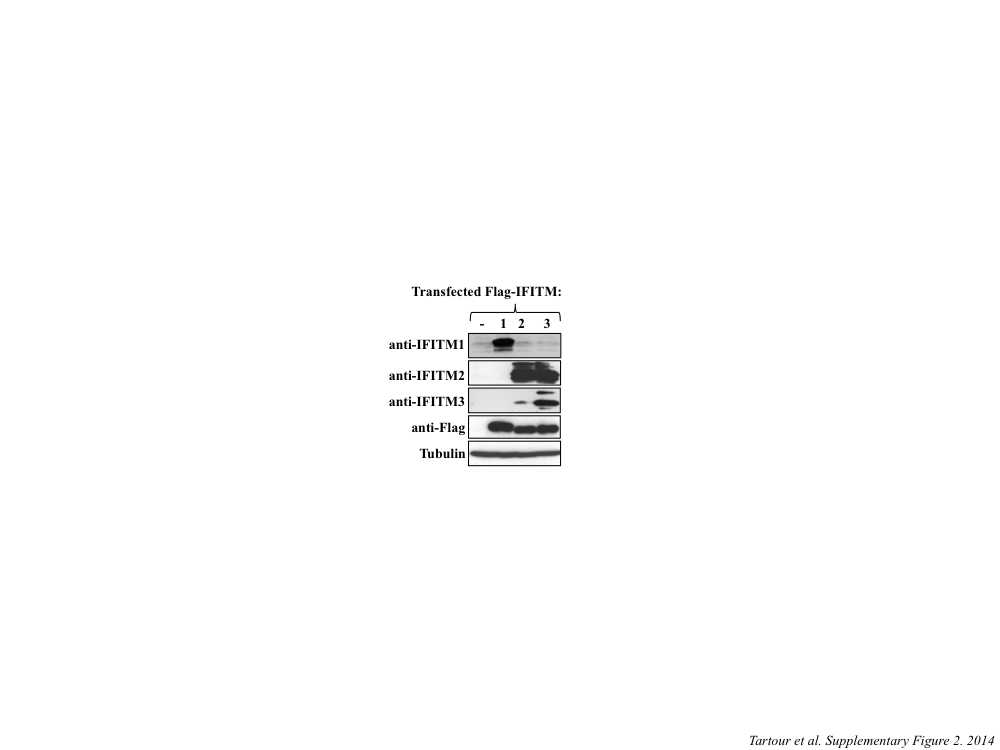

Supplement: Additional file 2: Figure S2. — Extent of cross-reactivity of commercially available anti-IFITM antibodies. To assess the specificity of commercial antibodies in our hands for the different IFITMs, HEK293T cells were transfected with each IFITM and cell lysates were then analyzed by WB. [file 12977_2014_103_MOESM2_ESM.tiff]
